# Supplementary material for: Stepped care, stepped care “lite” & matching intervention components to individual mental health needs: A rapid scoping review of mental health and substance use interventions for post-secondary students
Source: PLoS One. 2025 Mar 25;20(3):e0319473. doi: 10.1371/journal.pone.0319473 (PMC11936172; doi:10.1371/journal.pone.0319473)
Supplement: S1 Table — (DOCX) [file pone.0319473.s001.docx]

| **Lead Author of Study** | **Intervention Name** | **Mental Health issues addressed** | **Student Population** | **Study Design** | **N included in analysis** | **Primary Mental Health Outcome Measures** |
| --- | --- | --- | --- | --- | --- | --- |
| Farrer 2019^43^ | UniVirtual Clinic | Transdiagnostic (Depression, anxiety, and psychological distress) | Students ≥ 18 years, and score <15 on Kessler Distress Scale | Two-armed RCT; wait list control | 200 | -PHQ-9, GAD-7, Social Phobia Screener (SOPHS), Kessler Distress scale (K-10) |
| Fassnacht 2022^49^ | Be Well Plan Program | Distress | All students | RCT - parallel arm, waitlist control, unblinded | 164 | -PHQ-9; GAD-7 |
| Levin 2019^44^ | ACT Daily | Transdiagnostic (Depression and anxiety) | All but 1 participant was a student | RCT - Parallel arm - participants were partially blinded to condition assignment | 69 | -DASS-21; Mental Health Continuum Short Form |
| Haeger 2022^45^ | ACT Daily - self-guided | Transdiagnostic (depression & anxiety) | College students on wait list for counselling services with presenting problem of depression and/ or anxiety | Quasi-experimental Pre-post design | 11 | -DASS-21 |
| Bingham 2010^77^ | Michigan Prevention and Alcohol Safety for Students (M-PASS) | Alcohol use | First year students from 18 to 20 years old. | RCT: parallel arm | 1137 | -DDQ, Timeline Followback (TLFB), tolerance of drinking, Drinking Motives Questionnaire (DMQ), reasons not to drink, campus alcohol survey (CAS), attitudes towards drinking and driving scale (ADDS); drinking consequences (Young Adult Alcohol Problems Screening Test); riding with a drink/driver |
| Canale 2015^60^ |  | Alcohol use | Undergraduate and graduate students from psychology classes | Quasi-experimental eg. Pre-post | 124 | AUDIT; Drinking Motives Questionnaire Revised Short Form |
| Chiauzzi 2005^61^ | MyStudentBody.com: Alcohol (MSB: Alcohol) | Alcohol use | Undergraduate binge drinkers | RCT: Single blind | 265 | -DDQ, Alcohol Timeline Followback method; Rutgers Alcohol Problem Index (RAPI). |
| Essa-Hadad 2015^42^ |  | Smoking | Arab students attending colleges and universities | Quasi-experimental; Pre-post, with a qualitative arm | 225 | -Self-reporting cigarette and nargila smoking behaviour, intention to quit smoking, reason for wanting to quit, seeking of professional help to quit |
| McCloud 2020^67^ | Feel Stress Free | Depression; Anxiety | University students who scored 8+ on at least one subscale of the Hospital Anxiety and Depression Scale (HADS) | RCT: 6-week, web-based, parallel group, unblinded randomized controlled trial, with a wait-list control. Participants were individually randomized in a 1:1 ratio. | 168 | -Hospital Anxiety and Depression Scale (HADS) |
| Bruehlman-Senecal 2020^68^ | Nod | Depression & anxiety | First year students, 18-25 not living with parents | Pilot RCT; control group offered app after 4 week waiting period | 221 | -PHQ-9, GAD-7, Mini Social Phobia Inventory |
| Witkiewitz 2014^72^ | BASICS-Mobile | Alcohol use & smoking | Non-treatment seeking students | RCT: Participants were randomized to one of three conditions: BASICS-Mobile, daily monitoring only via mobile assessment, or minimal assessment control | 94 | -DDQ, Youth Adult Alcohol Problems Screening Test (YAAPST), Daily Smoking Questionnaire (DSQ) |
| Weitzel 2007^75^ |  | Alcohol use | Undergraduate | RCT: One control group and two treatment groups | 40 | -Timeline followback method for drinking behaviour, The Alcohol Consequences Expectancies Scale (ACES) and the Alcohol Consequence Self-Efficacy Scale (ACSES) |
| Voogt 2013^62^ | What Do You Drink (WDYD) | Alcohol use | Heavy drinkers 18-24 years old | RCT: two-group, parallel arm, no intervention control group | 907 | -AUDIT, heavy drinking, frequency of binge drink, weekly alcohol consumption using Alcohol Weekly Recall |
| Kazemi 2020^63^ | SmarTrek | Alcohol use | Mandated students (study 1); undergraduate psychology students who drank in the last month & owned iPhone (study 2) | RCT: parallel arm | 141 mandated 164 non-mandated | -AUDIT, DDQ, YAACQ |
| Lewis 2018^76^ |  | Alcohol use | Community college students aged 18-29 who binge drink or exceed weekly drinking recommendations | RCT | 48 | -DDQ |
| Gajecki 2014^64^ | PartyPlanner | Alcohol use | Students on student union email list | RCT: Parallel arm, unblinded | 1361 | -DDQ, AUDIT, blood alcohol level, binge drinking |
| Fitzsimmons-Craft 2020^37^ | StudentBodies - Eating Disorders (SB-ED) | Eating disorder | Female university students who screened positive for eating disorder (except anorexia nervosa) | RCT: Cluster | 690 | -PHQ-9, Patient-Reported Outcomes Measurement Information System anxiety short-form -EDE-Q; abstinence from ED behaviours; clinical impairment assessment |
| Jacobi 2012^38^ | StudentBodies+ | Eating disorder | Women aged 18-35 with subthreshold eating disorders | RCT: double blind | 126 | -BDI, EDE-Q; ED diagnostic section of the SCID number of daily meant, foods consumed and avoided; |
| Müssener 2016^73^ | NEXit | Smoking | Students who were daily or weekly smokers willing to set a quit date | RCT: single blind, 2 arm | 1502 | -self-reported prolonged abstinence, self-reported 4 week point prevalence of complete smoking cessation; self-reported, 7-day point prevalence of smoking abstinence; mean number of quit attempts since taking part in the study; number of uses of other smoking cessation services; the number of cigarettes smoked weekly among participants still smoking at the time of follow-up |
| Rahmadiana 2021^46^ | I-AiMentalWELLness (Saya menuju mental sehat) | Transdiagnostic (depression & anxiety) | Undergraduate & graduate students ≥18 years with mild to moderate depression or anxiety or both | Feasibility study | 96 | -PHQ-9, GAD-7 |
| Karyotaki 2022^47^ | ICare Prevent | Transdiagnostic (depression & anxiety) | >4 on PHQ9 or GAD7 | RCT: parallel arm, researchers blinded | 100 | -Mini-International Neuropsychiatric Interview; PHQ-9; GAD-7 |
| Obermayer 2004^74^ |  | Smoking | 18-25 college student with phone and smoked at least 28 cigarettes per week |  | 46 | Nicotine-Dependence Syndrome Scale; 7-day smoking-reconstruction form |
| Ohlmer 2013^39^ | StudentBodies - AN | Eating disorder (Anorexia Nervosa) | Women | Quasi-experimental | 32 | -BDI, Body Mass Index, WCS, EDE, EDI, Brief symptom inventory; |
| Richards 2016^69^ | Calming Anxiety | Anxiety | Scoring >10 on GAD7 | RCT: parallel arm, waitlist control | 137 | -BDI; GAD-7; Penn State Worry Questionnaire |
| Salamanca-Sanabria 2020^70^ | Yo puedo sentirmebien/I can feel better | Depression | ≥18 years, mild to moderate depression (score of 10-19 on PHQ-9) | RCT: Randomly assigned to either the treatment group or a waiting list control group. | 214 | -PHQ-9, GAD-7 |
| Winzelberg 2000^40^ | StudentBodies | Eating Disorders | Women with desire to improve body image satisfaction | RCT: unblinded, parallel arm |  | -Body shape Questionnaire (BSQ), EDI, EDE-Q |
| Escoffery 2004^65^ | Kick It! | Smoking | All students who smoke | RCT: pre-test, post-test & control group | 35 | -age of first cigarette, number of cigarettes per day, number of quit attempts |
| Atik 2023^71^ | elona therapy | Depression; Anxiety; | University students with mild to moderate symptoms of depression, or anxiety | Prospective uncontrolled trial | 100 | -PHQ-9, BDI, GAD-7, Beck Anxiety Inventory (BAI), Mental Health Literacy Scale (MHLS) |
| Benjet 2023^48^ | Space From Anxiety and Depression program (culturally adapted) | Depression; Anxiety; | Undergraduates at 7 universities in Colombia and Mexico, aged 18+, with clinically significant anxiety (GAD-7 ≥10) and/or depression (PHQ-9 ≥10) | Assessor-blinded, multisite, randomized clinical trial | 1,319 | -PHQ-9, GAD-7, Patient Health Questionnaire Anxiety and Depression Scale (PHQ-ADS) |
| Bohrer et al 2023^66^ | BEST-U | Eating Disorders | -university students ≥ 18 years with non-low-weight eating disorder diagnosis, access to a smartphone; and no uncorrected vision problems that would interfere with ability to participate in the study | Non-concurrent multiple-baseline design | 8 | -Eating Pathology Symptoms Inventory (EPSI), Clinical Impairment Assessment (CIA) |
