# Supplementary material for: Stepped care, stepped care “lite” & matching intervention components to individual mental health needs: A rapid scoping review of mental health and substance use interventions for post-secondary students
Source: PLoS One. 2025 Mar 25;20(3):e0319473. doi: 10.1371/journal.pone.0319473 (PMC11936172; doi:10.1371/journal.pone.0319473)
Supplement: S1 File — (DOCX) [file pone.0319473.s003.docx]

| 1. | exp Mental Disorders/ |  |  |  |  |
| --- | --- | --- | --- | --- | --- |
| 2. | exp Mental Health/ |  |  |  |  |
| 3. | exp Mental Health Services/ |  |  |  |  |
| 4. | ((mental* or psychol* or psychi*) adj2 (health or disorder* or condition* or ill or disease* or illness* or disabilit* or problem* or challeng*)).tw,id. |  |  |  |  |
| 5. | ((mental* or psychol* or psychi*) adj2 (stress* or distress* or well or wellness or wellbeing or well-being)).tw,id. |  |  |  |  |
| 6. | ((post-secondary or postsecondary or postgraduate* or graduate or graduates or undergrad* or under-grad* or doctora* or PHD or master or masters or academ* or cegep or tertiary) adj3 (student* or junior or juniors or freshman or freshmen or sophomore or sophomores or senior or seniors or campus* or cohort* or education* or program* or population* or course or courses or seminar or seminars)).tw,id. |  |  |  |  |
| 7. | (education* institution* or universit* or college* or polytechnic* or cegep).tw,id. |  |  |  |  |
| 8. | ((higher learning adj2 institution*) or (higher education* adj2 institution*)).tw,id. |  |  |  |  |
| 9. | ((intervention* or treatment* or therap* or service* or program* or support or care or approach* or platform* or app or app-based) adj4 (open access or tailor* or adapt or adapted or adaptability or adapts or adapting or adjusts or adjust or adjusted or adjusting or modif* or self-guid*)).tw,id. |  |  |  |  |
| 10. | ((strategy or strategies or activit* or module* or multi-component* or component* or level or levels or step or steps or stepped or need or needs or preference* or information or characteristic*) adj4 (tailor* or suited or suit or suits or fit or fits or choice or choose* or select* or open access or adapt or adapted or adaptability or adapts or adapting or adjusts or adjust or adjusted or adjusting or modif*)).tw,id. |  |  |  |  |
| 11. | (stepped care or stepped-care or clinical staging).tw,id. |  |  |  |  |
| 12. | 1 or 2 or 3 or 4 or 5 |  |  |  |  |
| 13. | 6 or 7 or 8 |  |  |  |  |
| 14. | 9 or 10 or 11 |  |  |  |  |
| 15. | 12 and 13 and 14 |  |  |  |  |
